# Supplementary material for: Towards reliable extreme weather and climate event attribution
Source: Nat Commun. 2019 Apr 15;10:1732. doi: 10.1038/s41467-019-09729-2 (PMC6465259; doi:10.1038/s41467-019-09729-2)
Supplement: Supplementary file 1 — Supplementary Information [file 41467_2019_9729_MOESM1_ESM.pdf]

Supplementary Information

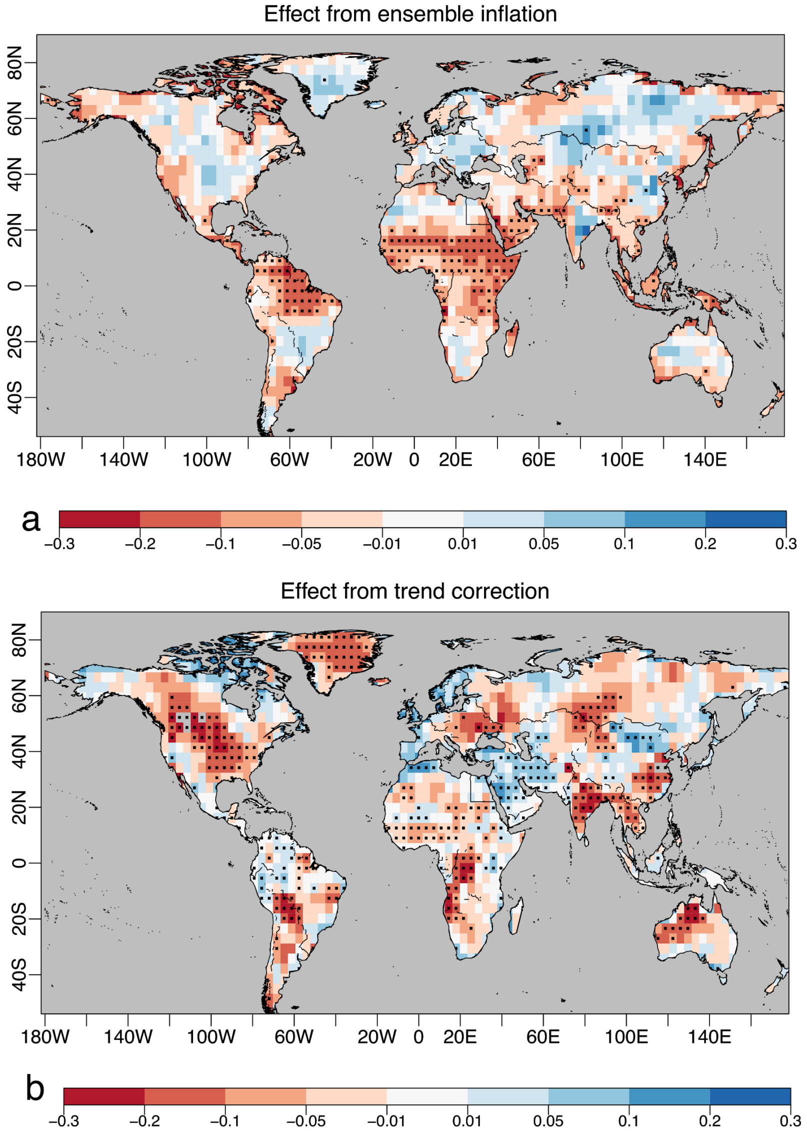

Supplementary Figure 1: Influence of the ensemble correction and the long-term trend correction on the change in the fraction of attributable risk (FAR). The two factors are separated for the analysis illustrated in figure 3. The separation is obtained by calibrating either only the ensemble spread and ensemble mean anomaly (first panel) or by correcting the long-term trend alone (second panel).

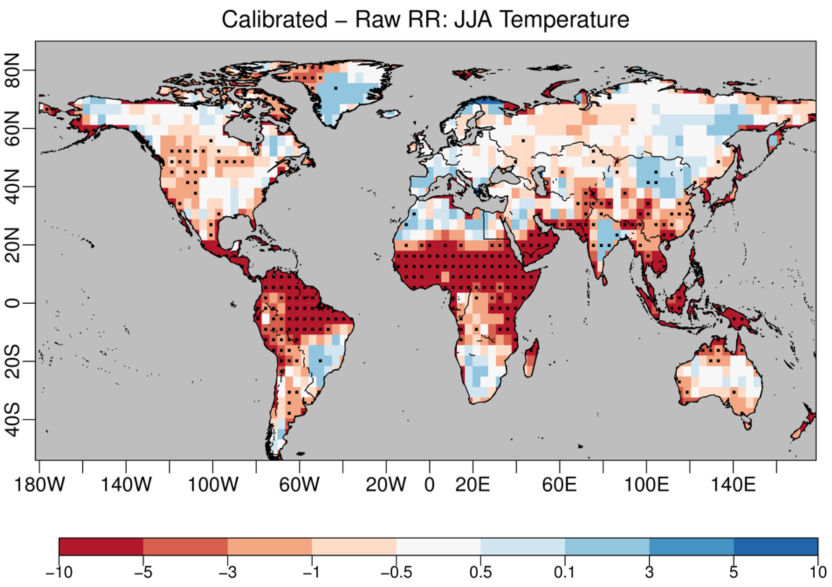

Supplementary Figure 2: Influence of the ensemble correction as shown in figure 3 but here for the risk ratio (RR) instead of FAR.

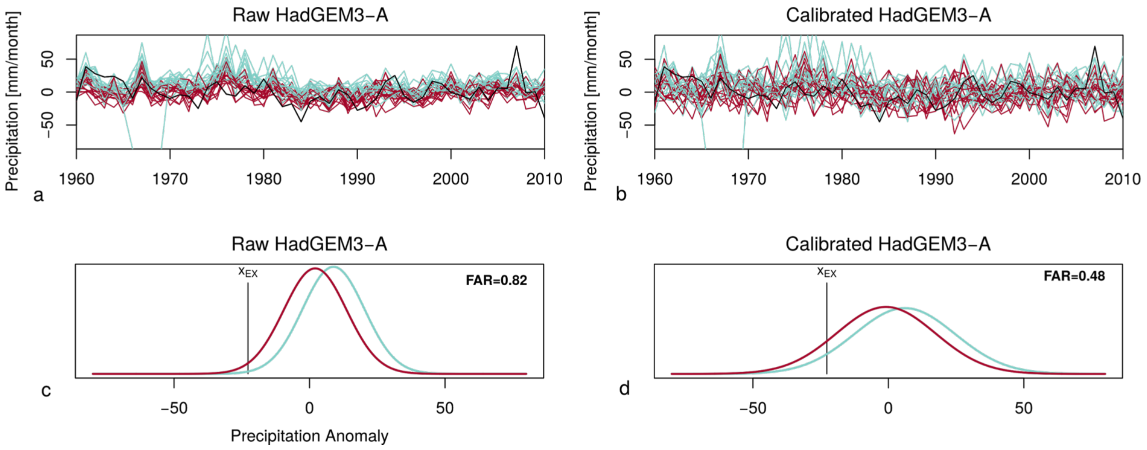

Supplementary Figure 3: Effect of the calibration on the fraction of attributable risk (FAR) to climate change on an example of dry summers. The same analysis as in Fig. 1 (Sudan, 12.6 N, 34.4 W) but for summer (June-to-August) mean precipitation anomalies and observations from GPCC[53]. The impact on FAR is calculated using a negative precipitation anomaly (a dry summer) which would occur in the observations 1 in 10 years. The impact on FAR is stronger in this example due to the weaker mean shift between the two distributions (associated with the climate change forcing) for precipitation changes.

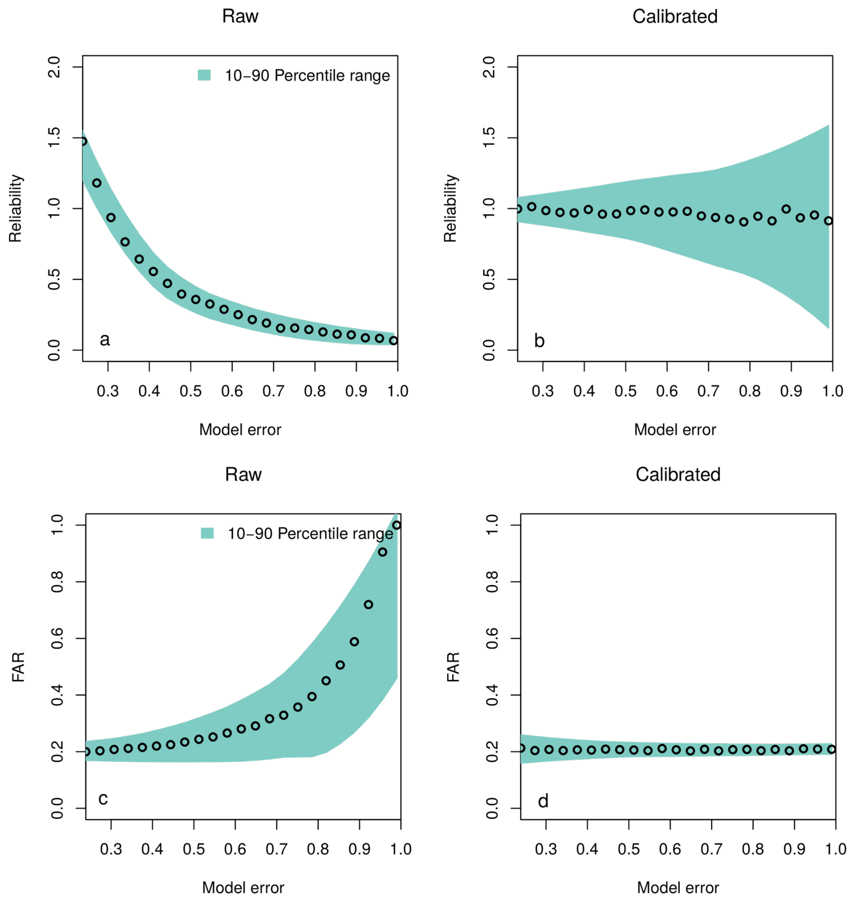

Supplementary Figure 4: Application of the proposed calibration procedure on on a statistical model where the conditional model error can be altered and the true value of FAR can be fixed (0.2 in this example)[12]. While increasing the model error reduces the reliability and increases FAR[12], the calibrated model corrects the reliability and perfectly reproduces FAR prescribed in the model.
